# Efficacy of dupilumab plus topical corticosteroids in children with atopic dermatitis: A meta‐analysis of randomized controlled trials

**DOI:** 10.1002/iid3.1133

**Published:** 2024-01-10

**Authors:** Li Wang, Si‐Ning Wang, Ruil‐Li Zhang

**Affiliations:** ^1^ The Second Affiliated Hospital of Nanjing Medical University Nanjing Jiangsu Province China

**Keywords:** atopic dermatitis, children, corticosteroids, dupilumab

## Abstract

**Introduction:**

Children with atopic dermatitis (AD) bear a significant burden of illness that adversely affects their quality of life.

**Objective:**

To determine the efficacy of dupilumab and topical corticosteroids for the treatment of pediatric AD.

**Methods:**

A comprehensive literature search was conducted using three prominent databases: Web of Science, PubMed, and Embase. Using a fixed‐effects or random‐effects model, the standard mean difference or risk ratios with 95% confidence intervals were calculated, and the trial protocol was listed as CRD42023408546.

**Results:**

A total of 3 studies were included, and 896 participants met the inclusion criteria. The combined estimate showed that dupilumab plus topical corticosteroids had numerically greater efficacy in terms of Eczema Area and Severity Index (EASI)‐50, EASI‐75, EASI‐90, and Investigator Global Assessment (IGA) score of 0 or 1. Children who received topical corticosteroids and dupilumab achieved significantly higher Children's Dermatological Life Quality Index scores compared to those who received placebo. The number of individuals who achieved IGA 0/1 increased with the use of dupilumab and topical corticosteroids.

**Conclusions:**

Dupilumab and topical corticosteroids can be used to treat symptoms in children with AD. However, given the substantial variation in treatment outcomes among studies, the findings should be interpreted with caution.

## 1 INTRODUCTION

The pathophysiology of atopic dermatitis (AD) is complex and multifactorial, involving alterations in lipid composition, immunological diseases, skin barrier malfunction, mutations in epidermal genes, and microbial imbalance.^1^ Affecting up to 20% of children worldwide, AD is a condition with a complex etiology, as evidenced by research.^2^, ^3^ A multistep strategy is needed to achieve therapeutic goals, focusing on reducing pruritus and achieving disease control. The primary drugs used to treat AD in children include topical emollients, calcineurin inhibitors, glucocorticoids, oral antihistamines, and so forth.^4^ Dupilumab is a human IgG4 monoclonal antibody that blocks interleukin (IL)‐4 and IL‐13 signaling, thereby reducing the molecular mechanisms underlying type 2 inflammatory diseases.^5^ Multiple clinical trials have demonstrated the effectiveness of dupilumab plus topical corticosteroids for AD in children. However, the evidence is not yet strong enough to make a solid judgment owing to the small sample size, variable results, and varied dosages used in the trials.^6^

Therefore, we conducted a meta‐analysis to evaluate the efficacy of topical corticosteroids in combination with dupilumab in young patients with AD.

## 2 METHODS

The study protocol was registered with PROSPERO under identification number CRD42023408546.

### 2.1 Search strategy

Adhering to the Preferred Reporting Items for Systematic Reviews and Meta‐Analyses (PRISMA) guidelines,^7^ a comprehensive literature search was conducted to identify relevant studies evaluating the efficacy of dupilumab combined with topical corticosteroid treatment in children with AD. Two independent reviewers thoroughly searched the literature for relevant studies using the terms dermatitis, AD, children, and dupilumab in conjunction with randomized controlled trials (RCTs). The search encompassed three prominent electronic databases (PubMed, Web of Science, and Embase), focusing on studies published between 2020 and 2022. Taking PubMed as an example, the search strategy is as follows。

#1“Dermatitis, Atopic”[Mesh]

#2 (Atopic Dermatides[Title/Abstract]) OR (Atopic Dermatitis[Title/Abstract])) OR (Dermatitides, Atopic[Title/Abstract])) OR (Neurodermatitis, Atopic[Title/Abstract])) OR (Atopic Neurodermatitides[Title/Abstract])) OR (Atopic Neurodermatitis[Title/Abstract])) OR (Neurodermatitides, Atopic[Title/Abstract])) OR (Neurodermatitis, Disseminated[Title/Abstract])) OR (Disseminated Neurodermatitides[Title/Abstract])) OR (Disseminated Neurodermatitis[Title/Abstract])) OR (Neurodermatitides, Disseminated[Title/Abstract])) OR (Eczema, Atopic[Title/Abstract])) OR (Atopic Eczema[Title/Abstract])) OR (Eczema, Infantile[Title/Abstract])) OR (Infantile Eczema[Title/Abstract])

#3#1 OR #2

#4“Children”[Mesh]

#5(children[Title/Abstract])) OR (child [Title/Abstract])

#6#4 OR #5

#7(Dupilumab[Mesh])

#8#3 AND #6 AND #7

### 2.2 Selection criteria

The publications were considered for inclusion in this meta‐analysis if they met the following criteria: (1) The studies involved children under 12 years old, who met the diagnostic criteria for moderate to severe AD, had an insufficient response to local treatment, or were unsuitable for local treatment; (2) Dupilumab was used as the intervention therapy; (3) Placebo or any other therapy could be used as the control therapy; (4) The study reported at least one of the following outcomes: an improvement in the Children's Dermatology Life Quality Index (CDLQI), an improvement in the Eczema Area and Severity Index (EASI)‐90, an improvement in the EASI‐75, and an improvement in the Investigator's Global Assessment (IGA); (5) RCTs were conducted.

### 2.3 Data extraction

A customized data extraction sheet was used to retrieve data from early trials. Every data point was independently entered twice, by two different people, to minimize data entry errors. Any discrepancies were discussed and resolved during a group meeting. The following information was extracted from the included studies whenever available: first author, country, year of publication, study design, sample size, follow‐up period, patient characteristics (inclusion criteria, mean age, dupilumab dosage, and outcome measures), and study characteristics (first author, follow‐up period).

### 2.4 Risk of bias assessment

The Cochrane Handbook for Systematic Reviews of Interventions Risk of Bias Tool was used to assess the caliber of the included RCTs. This tool assess various types of bias, including incomplete outcome data (attrition bias), selective reporting of results (reporting bias), blinding of participants and staff (performance bias), blinding of outcome assessment (detection bias), and others. The risk of bias assessments ranged from high to low to unclear. The trial was rated as having a high risk of bias if any aspect of randomization or blinding was deemed high‐risk. The authors reached an agreement to avoid any misunderstanding. Statistical significance was evaluated using the Egger's regression test, and publication bias was evaluated using funnel plots and statistical analyses.

### 2.5 Statistical analysis

All statistical evaluations were performed using RevMan 5.4. Relative risk and 95% confidence interval (CI) were used to determine efficacy and safety indicators. Interstudy heterogeneity was assessed using *Q* and *I* ^2^ tests. Fixed‐effect models were used when the heterogeneity was minimal (*p* > .1, *I* ^2^ ≤ 50%); random‐effects models were used when the heterogeneity was substantial (*p* < .1, *I* ^2^ > 50%); and sensitivity analyses were performed for instances where heterogeneity was evident. Statistical significance was set at *p* < .05.^8^, ^9^, ^10^

## 3 RESULTS

### 3.1 Study selection

**Figure 1:**
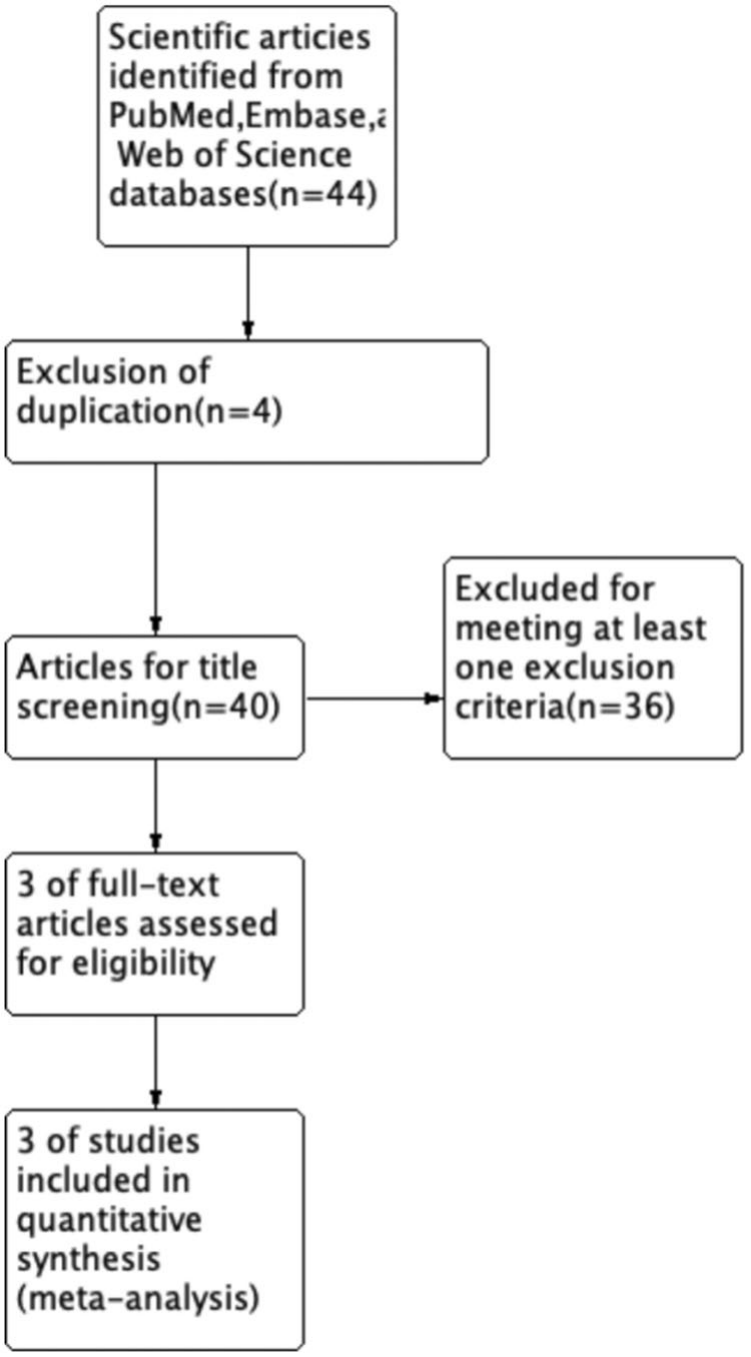
Eligibility of studies for inclusion in meta‐analysis.

An initial search yielded a total of 380 publications. After excluding studies that did not meet the inclusion and exclusion criteria (animal experiments, reviews, non‐RCTs, non‐Chinese or English language, no defined drug, etc.). Three RCTs were added. The general features of the eight RCTs are presented in Table 1. No additional publications from previous studies were found (Figure 1).^11^, ^12^, ^13^

**Table 1:** The general features of the three trials.

Study	Year of publication	Inclusion criteria	Diagnostic criteria	Interventions		Outcome measures
				Experimental group	Control group	
Paller AS	2020	IGA ≧ 3	AADCC	Dupilumab + TGC:100/200 mg q2w (122); 300 mg q4w (122)	Placebo + TGC (123)	①②③④
Alan D. Irvine	2021	IGA ≧ 3	AADCC	Baseline weight < 30 kg:Dupilumab 300 mg q4w + TCS (*n* = 61); Baseline weight ≥ 30 kg:Dupilumab 200 mg q2w + TCS (*n* = 59)	Baseline weight < 30 kg:placebo + TGC (61); Baseline weight ≥ 30 kg:placebo + TGC (59)	②④⑤
Paller AS	2022	IGA ≧ 3	AADCC	Dupilumab + TGC (83); 共16周	Placebo + TGC (79)	①②③④⑤

*Note*: (1) IGA; (2) EASI; (3) Atopic dermatitis score (SCORAD); (4) NRS score;(5) DLQI score.

Abbreviations: AADCC, American Academy of Dermatology consensus criteria; DLQI, Dermatology Life Quality Index; EASI, Eczema Area and Severity Index; IGA, Investigator Global Assessment; NRS, Numerical Rating Scale; q2w, once every 2 weeks; q4w, once every 4 weeks; qw, once a week; TCS, topical corticosteroids; TGC, topical glucocorticoids were used.

### 3.2 Study parameters and the possibility of bias

**Figure 2:**
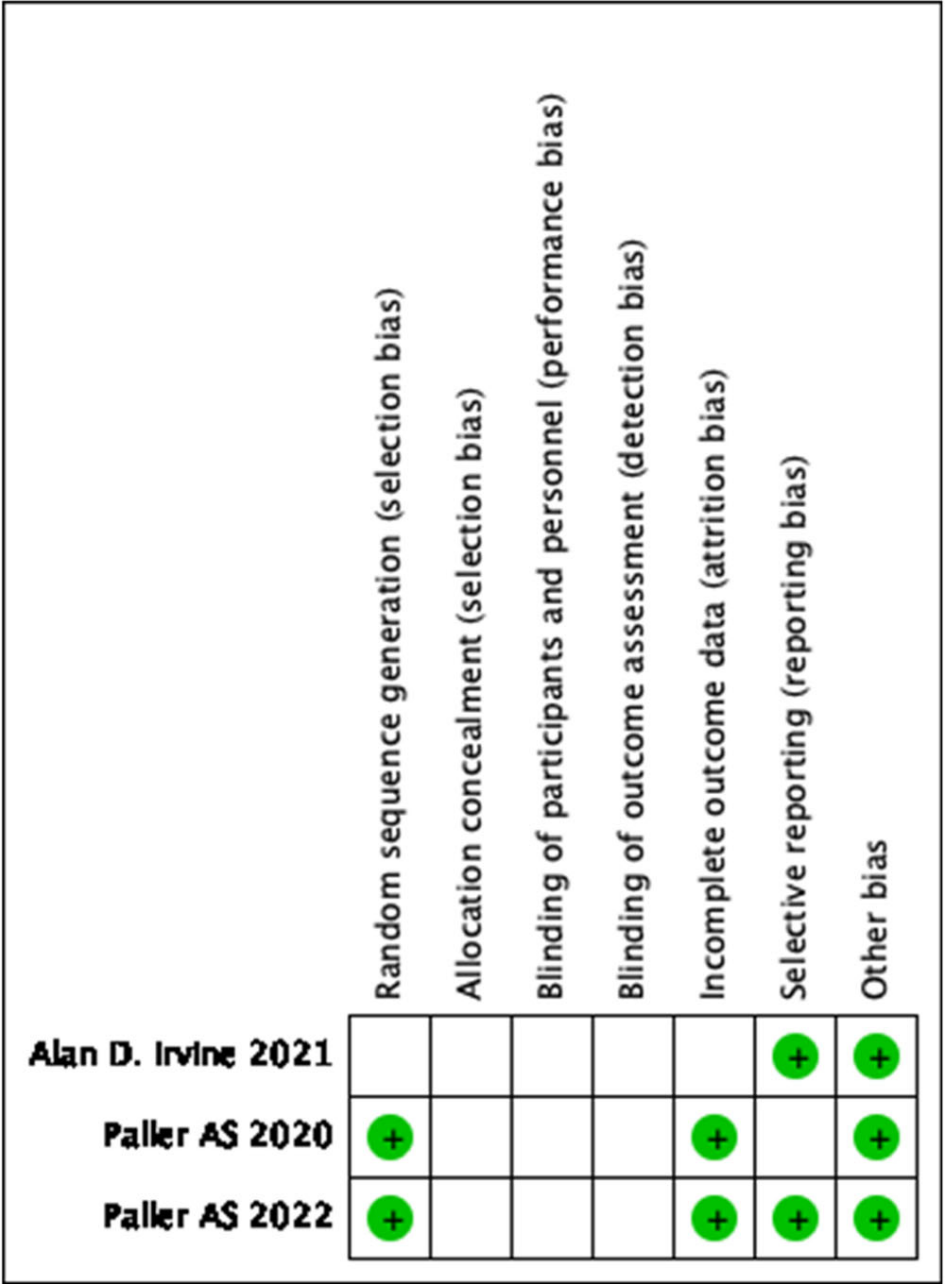
Risk of bias summary.

All included studies were published between 2020 and 2022. Figure 2 shows the risk of bias assessment. All studies were randomized, double‐blind, and placebo/placebo + topical corticosteroids‐controlled. None of the studies described a specific allocation concealment scheme, and the implementation of blinding was rated as unclear. All studies were evaluated as low‐risk because no evidence of damage to data integrity or selective reporting was found, and no other factors causing bias were identified. None of the studies described a specific allocation concealment scheme, and the implementation of blinding was therefore rated as unclear.

### 3.3 IGA responders (IGA 0/1)

**Figure 3:**
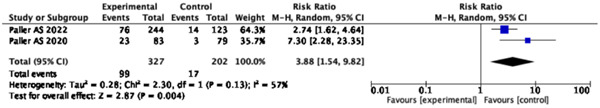
Forest plot of effect of dupilumab plus topical corticosteroids treatment on Investigator Global Assessment scores in atopic dermatitis patients.

According to the pooled data, topical corticosteroids added to dupilumab significantly increased the number of IGA responders compared to placebo (MD = 3.88; 95% CI: [1.54, 9.82, *p* = .004]) (Figure 3).

### 3.4 EASI‐75 responders

**Figure 4:**
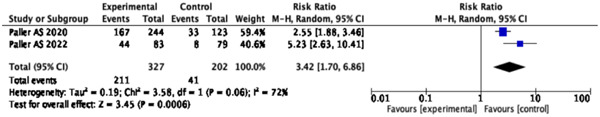
Forest plot of effect of dupilumab plus topical corticosteroids treatment on Eczema Area and Severity Index‐75 in atopic dermatitis patients.

Comparing placebo to dupilumab plus topical corticosteroids, the pooled analysis showed that the latter considerably boosted EASI‐75 responders (MD = 3.42; 95% CI: [1.70, 6.86], *p* = .0006) (Figure 4).

### 3.5 EASI‐50 responders

**Figure 5:**
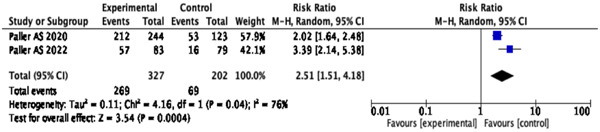
Forest plot of effect of dupilumab plus topical corticosteroids treatment on Eczema Area and Severity Index‐50 in atopic dermatitis patients.

According to the pooled analysis, dupilumab plus topical corticosteroids significantly increased the number of EASI‐50 responders compared to the placebo (MD = 2.51; 95% CI: [1.51, 4.18], *p* = .0004) (Figure 5).

### 3.6 EASI‐90 responders

**Figure 6:**
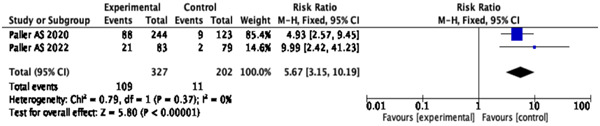
Forest plot of effect of dupilumab plus topical corticosteroids treatment on Eczema Area and Severity Index‐90 in atopic dermatitis patients.

According to the pooled analysis, dupilumab plus topical corticosteroids significantly enhanced EASI‐90 responders compared to the placebo (MD = 5.67; 95% CI: [3.15, 10.19], *p* < .00001) (Figure 6).

### 3.7 CDLQI

Two studies that reported CDLQI results involved children with AD who received topical corticosteroids and dupilumab doses of 100/200 mg q2w and 300 mg q4w.^10^, ^11^ According to subgroup analysis based on the time interval and dose (100/200 mg q2w: standard mean difference [SMD] = −9.77, 95% CI: −17.55, −2.00; 300 mg q4w: SMD = −10.48, 95% CI: −18.82, −2.15, *p* = .01), dupilumab significantly reduced the change in CDLQI score compared to other therapies.

**Figure 7:**
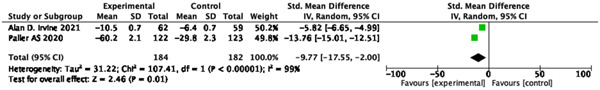
Forest plot of effect of 100/200 mg q2w dupilumab plus topical corticosteroids treatment on Children's Dermatology Life Quality Index in atopic dermatitis patients.

**Figure 8:**
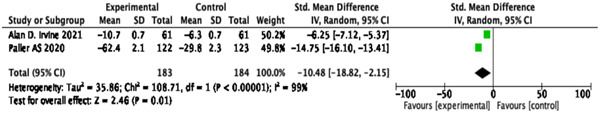
Forest plot of effect of 300 mg q4w dupilumab plus topical corticosteroids treatment on Children's Dermatology Life Quality Index in atopic dermatitis patients.

For 100/200 mg q2w dupilumab with topical corticosteroids against placebo and 300 mg q4w dupilumab plus topical corticosteroids versus placebo, the CDLQI is shown in Figures 7 and 8.

## 4 DISCUSSION

Topical glucocorticoids are often the first‐line treatment for children with AD; however, long‐term use of steroids over large areas of the skin can have adverse effects on the skin and other systems.^2^ Therefore, another viable treatment option is required for this population. Dupilumab has emerged as a promising drug for children with AD who have not responded adequately to conventional therapy.^14^

This meta‐analysis examined how well dupilumab and topical corticosteroids work together to treat AD in young patients. The percentages of individuals who saw improvements in the IGA response, the EASI‐75, the EASI‐50, and the EASI‐90 were examined in the analysis of three trials. Additionally, the positive influence of dupilumab on patient‐reported outcomes, such as the CDLQI, has been established. Numerous systematic reviews of trials conducted in adults have supported the effectiveness of dupilumab and concomitant topical corticosteroids in children.^15^ According to large sample clinical studies conducted abroad in 2017, dupilumab with concurrent topical corticosteroids might considerably lower overall disease severity in patients with AD and enhance pruritus and associated quality of life in patients.

This meta‐analysis represents the first comprehensive evaluation of the combined use of topical corticosteroids and dupilumab in the treatment of juvenile AD, both domestically and internationally. The findings hold promise for informing future diagnostic and treatment strategies for AD. However, this study has some limitations that should be acknowledged. First, to further investigate this issue, larger scale RCTs are warranted, surpassing the three RCTs included in this analysis. Second, most of the included trials were only 16 weeks long, rendering them unable to demonstrate long‐term efficacy. Finally, potential language bias may exist as only RCTs published in English were considered.

## 5 CONCLUSION

In conclusion, the combination of topical corticosteroids and dupilumab has demonstrated significant efficacy in alleviating clinical symptoms in children with AD. The aforementioned findings need to be validated by high‐quality, large‐sample, long‐term clinical trials owing to sample size, and follow‐up time limits.

## AUTHOR CONTRIBUTIONS

**Li Wang**: Conceptualization; methodology; formal analysis; writing—original draft; writing—review and editing; project administration. **Si‐Ning Wang**: Conceptualization; investigation; data curation; writing‐original draft. **Ruil‐Li Zhang**: Conceptualization; supervision; project administration.

## CONFLICT OF INTEREST STATEMENT

The authors declare no conflict of interest.
